# Biochemical Characterization of Pyranose Oxidase from *Streptomyces canus*—Towards a Better Understanding of Pyranose Oxidase Homologues in Bacteria

**DOI:** 10.3390/ijms232113595

**Published:** 2022-11-06

**Authors:** Anja Kostelac, Leander Sützl, Jolanta Puc, Valentina Furlanetto, Christina Divne, Dietmar Haltrich

**Affiliations:** 1 Laboratory of Food Biotechnology, Department of Food Science and Technology, BOKU-University of Natural Resources and Life Sciences, 1180 Vienna, Austria; 2 Doctoral Programme BioToP-Biomolecular Technology of Proteins, BOKU-University of Natural Resources and Life Sciences, 1180 Vienna, Austria; 3 School of Engineering Sciences in Chemistry, Biotechnology, and Health-CBH, KTH Royal Institute of Technology, SE-100 44 Stockholm, Sweden

**Keywords:** pyranose oxidase, glycosides, kinetics, structure, characterization, bacterial lignocellulose degradation, CAZy AA3

## Abstract

Pyranose oxidase (POx, glucose 2-oxidase; EC 1.1.3.10, pyranose:oxygen 2-oxidoreductase) is an FAD-dependent oxidoreductase and a member of the auxiliary activity (AA) enzymes (subfamily AA3_4) in the CAZy database. Despite the general interest in fungal POxs, only a few bacterial POxs have been studied so far. Here, we report the biochemical characterization of a POx from *Streptomyces canus* (*Sc*POx), the sequence of which is positioned in a separate, hitherto unexplored clade of the POx phylogenetic tree. Kinetic analyses revealed that *Sc*POx uses monosaccharide sugars (such as d-glucose, d-xylose, d-galactose) as its electron-donor substrates, albeit with low catalytic efficiencies. Interestingly, various C- and O-glycosides (such as puerarin) were oxidized by *Sc*POx as well. Some of these glycosides are characteristic substrates for the recently described FAD-dependent C-glycoside 3-oxidase from *Microbacterium trichothecenolyticum*. Here, we show that FAD-dependent C-glycoside 3-oxidases and pyranose oxidases are enzymes belonging to the same sequence space.

## 1. Introduction

The Carbohydrate-Active enZYme database (CAZy) (www.cazy.org) is a repository of catalytic modules and domains of enzymes that degrade, modify, or create glycosidic bonds. In conjunction to the CAZy enzymes, auxiliary activity (AA) enzymes provide a helping hand during lignocellulose degradation [1]. Currently, 17 families of auxiliary activity cover redox enzymes that act in conjunction with CAZymes, namely nine families of ligninolytic enzymes and eight families of lytic polysaccharide monooxygenases. The ligninolytic AA family 3 (AA3) exclusively includes members of the glucose–methanol–choline (GMC) superfamily of FAD-dependent oxidoreductases [2] and is structured into four subfamilies. Subfamily AA3_1 contains the flavodehydrogenase domain of cellobiose dehydrogenase; subfamily AA3_2 includes aryl alcohol oxidase, glucose oxidase, glucose dehydrogenase and pyranose dehydrogenase; subfamily AA3_3 consists of alcohol (methanol) oxidases and finally, subfamily AA3_4 comprises pyranose oxidases [3]. Pyranose oxidase (POx, glucose 2-oxidase; EC 1.1.3.10, pyranose:oxygen 2-oxidoreductase) is an FAD-dependent oxidoreductase [4] and has been studied extensively in the last 20 years mainly from fungal sources, both biochemically and structurally [5,6], because of potential bioelectrochemical and biocatalytic applications.

The reaction mechanism of POx involves two half-reactions: the reductive and the oxidative half-reaction. In the reductive half-reaction, two electrons are transferred as a hydride equivalent from the electron donor to the FAD, forming the reduced form of FADH_2_. In the oxidative half-reaction, FADH_2_ is reoxidized to its initial form while the electron acceptor is reduced. Preferred electron donor substrates for fungal POxs are various aldopyranoses (e.g., d-glucose, d-galactose, d-xylose and glucono-δ-lactone), while preferred electron acceptors include molecular oxygen, quinones (e.g., p-benzoquinone) and metal ions (e.g., the ferrocenium ion Fc^+^). Oxidation of aldopyranoses typically occurs at the C2 position, yielding a 2-ketopyranose, but can also take place at the C3 position, yielding a 3-ketopyranose [7]. A typical fungal POx monomer is comprised of an FAD-binding domain (a Rossmann domain of class α/β), and a substrate-binding domain (a six-stranded central β-sheet and associated α-helices). Fungal POxs form tetramers, where the access of the substrate to the active site is modulated by a large cavity and two access channels that allow the substrate molecule to enter the active site. Furthermore, its reactivity is fine-tuned by a mobile active-site loop [8]. The FAD moiety in fungal POx is covalently linked to a histidine residue, resulting in one FAD molecule per POx monomer.

**Figure 1:**
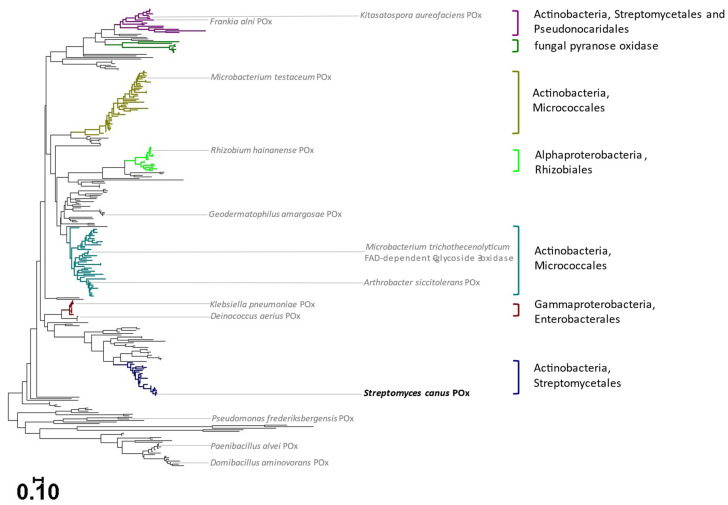
Phylogenetic tree of putative pyranose oxidase sequences, depicting the diversity of bacterial pyranose oxidases. Fungal sequences are represented by dark green branches. The positions of previously characterized POx from *Arthrobacter siccitolerans* and *Kitasatospora aureofaciens* are indicated in the tree as well as that of the FAD-dependent C-glycoside 3-oxidase from *Microbacterium trichothecenolyticum* (grey, right side of enzyme names). The position of POx from *Streptomyces canus*, which was characterized in this study, is shown in black, as are the positions of the nine sequences used in the initial screening for activity (grey, left side of enzyme names). Sequences from a characterized fungal cellobiose dehydrogenase and bacterial cholesterol oxidases were used as outgroups. The bar represents phylogenetic distance as amino acid substitution per site.

Pyranose oxidases have been characterized mainly from wood-degrading fungi, where they are secreted extracellularly and associated with membrane-bound vesicles or other types of structures of fungal hyphae [9]. To date, POxs of fungal origin were biochemically characterized from *Aspergillus oryzae, Aspergillus nidulans*, *Irpex lacteus*, *Lyophyllum shimeji*, *Peniophora gigantea*, *Peniophora* sp., *Phanerochaete chrysosporium*, *Phlebiopsis gigantea*, *Trametes multicolor* (*ochracea*) and *Tricholoma matsutake* [3,6]. Already, in 2009, it was hypothesized that *pox* genes first evolved in bacteria and were then introduced into fungi via horizontal gene transfer [10]. A newly constructed phylogenetic tree (Figure 1) confirms the close relationship between putative bacterial and fungal POx sequences, despite their sharing sequence similarities of only 26 to 39%. It also shows that putative bacterial POxs are mainly found in the phyla of Actinobacteria and Proteobacteria.

Pyranose oxidases of bacterial origin were studied only lately, and the first biochemically characterized enzymes originate from *Arthrobacter siccitolerans* (*As*POx) and *Kitasatospora aureofaciens* (previously known as *Streptomyces aureofaciens*) (*Ka*POx) [11,12]. In the POx phylogenetic tree, *Ka*POx is positioned in the clade closest to fungal POx sequences, whereas the sequence of *As*POx is positioned in a clade completely separated from these (Figure 1). Recently, the FAD-dependent enzyme C-glycoside 3-oxidase from *Microbacterium* sp. 5-2b (CarA) and related species (*Arthrobacter globiformis* and *Microbacterium trichothecenolyticum* (*Mt*CarA)) was reported to be closely related to bacterial POxs [13]. The sequence of *Mt*CarA is indeed found within a Micrococcales clade of pyranose oxidases, closely positioned to that of *As*POx (Figure 1). The crystal structure of *Mt*CarA was solved at 2.4-Å resolution (PDB accession ID 7DVE). Its structure shared similarity with that of a subunit of a fungal POx from *Peniophora* sp. (PDB 1TZL) with an rmsd value of 2.5 Å (454 Cα atoms; 28% amino acid sequence identity) [13]. In accordance with the fungal POx monomers, the structure of bacterial *Mt*CarA showed the typical FAD and substrate-binding domains.

Bearing in mind that only two bacterial pyranose oxidases have been characterized so far, we studied a not yet investigated bacterial POx in order to get a better insight into this class of enzymes. We selected the sequence of *Streptomyces canus* pyranose oxidase (*Sc*POx), which is positioned in a separate, hitherto unexplored POx clade containing sequences from Actinobacteria and Streptomycetales, as this would expand our knowledge of the sequence space of bacterial pyranose oxidases. Our study provides a detailed biochemical characterization of *Sc*POx, with the emphasis on substrate specificity, steady-state kinetic parameters and stability. Additionally, a comparison of the *Sc*POx structural model with other structures was performed.

## 2. Results

### 2.1. Screening and Expression of Different Bacterial Pyranose Oxidases

Ten bacterial POx sequences from various branches of the phylogenetic tree (Figure 1) were initially selected for screening. The sequences were from the following organisms: *Frankia alni, Paenibacillus alvei, Domibacillus aminovorans, Klebsiella pneumoniae, Rhizobium hainanense, Deinococcus aerius, Microbacterium testaceum, Streptomyces canus, Geodermatophilus amargosae* and *Pseudomonas frederiksbergensis.* The expression of the corresponding genes upon induction with lactose in *E. coli* yielded proteins of the expected size (analysis by SDS-PAGE, protein bands of 50 to 65 kDa), and the presence of the His-tag was confirmed by Western blotting. When testing for activity with d-glucose as electron acceptor substrate, only two of the recombinant enzymes showed prominent activity levels—POx from *Deinococcus aerius* and *Streptomyces canus* (*Sc*POx). The enzyme *Sc*POx was selected for further characterization.

After a two-step purification process based on affinity and size-exclusion chromatography, the expression yielded ~5 mg of apparently pure *Sc*POx per 1 L of *E. coli* culture. SDS-PAGE analysis confirmed the purity of the enzyme preparation and was in good agreement with the theoretical mass of 52 kDa (Supplementary Materials Figure S1). Analysis by size exclusion chromatography resulted in a molecular mass of 52.4 ± 1.0 kDa (Supplementary Materials Figure S2), indicating that *Sc*POx, in contrast to fungal POx, is monomeric in solution. Furthermore, the purified recombinant protein showed the typical bright yellow colour of flavoproteins. MALDI-TOF analysis of the yellow supernatant after TCA precipitation of *Sc*POx revealed the theoretical mass of FAD (785.5 kDa), indicating that *Sc*POx is indeed an FAD-containing enzyme and that the prosthetic group is noncovalently bound.

**Figure 2:**
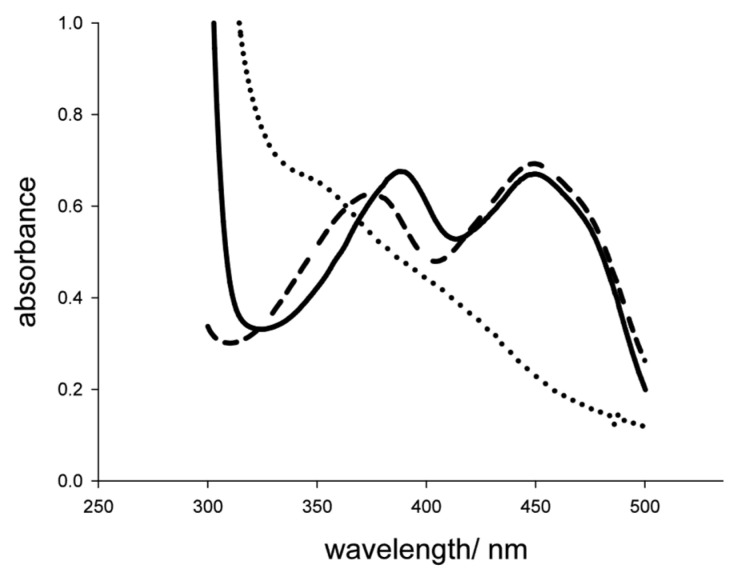
UV-Vis spectra of *Sc*POx in its oxidized form (solid line), after addition of 4 M d-xylose under aerobic conditions (dotted line) and of the supernatant after TCA precipitation (dashed line).

UV-Vis spectra of *Sc*POx showed the characteristic FAD peaks with maxima at 390 nm and 450 nm. These two peaks disappeared upon reduction of the enzyme with 4 M d-xylose, which is a characteristic for FAD-dependent oxidoreductases (Figure 2). Release of the prosthetic group after TCA treatment showed a blueshift of the flavin peaks, again indicating a noncovalent association of FAD with the polypeptide. The FAD occupancy, as calculated from the molar extinction coefficient of the enzyme (ε_280_ = 10,928 M^−1^ cm^−1^) and released free FAD, was determined to be ~100%. This means that one FAD molecule is bound to one monomer of the enzyme.

### 2.2. Kinetic Characterisation

We determined the apparent steady-state kinetic constants for different electron acceptors, namely, p-benzoquinone (p-BQ), 2,6-dichlorophenolindophenol (DCIP) and oxygen (air saturation), with d-xylose as electron donor. The enzyme showed pronounced dehydrogenase activity with p-BQ and DCIP and rather low oxidase activity. The ferrocenium ion Fc^+^, which was found to be a good electron acceptor for *Ka*POx, did not exhibit any activity with *Sc*POx.

**Figure 3:**
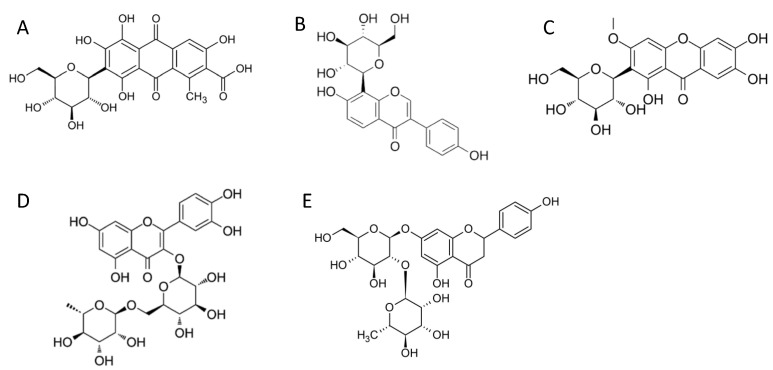
Structure of C- and O-glycosides used as substrates by *Sc*POx. (**A**) carminic acid; (**B**) puerarin; (**C**) mangiferin; (**D**) rutin and (**E**), naringin.

Initial experiments gave surprisingly low activities with d-glucose, given that it is the most typical POx substrate, hence a range of different sugars and sugar alcohols were tested as possible preferred substrates of *Sc*POx (Table 1). *Sc*POx was found to be active on d-glucose, d-galactose, d-xylose, d-ribose and l-arabinose, with d-glucose showing the highest activity (0.045 U/mg), and no activity was detected with l-lyxose, l-sorbose, d-arabinose, d-mannose, d-fructose, maltose, trehalose, lactose, sucrose, cellobiose, d-mannitol or xylitol. Given that a C-glycoside 3-oxidase was recently shown to be closely related to POx [13], various C- and O-glycosides were also tested as possible substrates (structures in Figure 3), and indeed *Sc*POx showed substantial activity with the C-glycoside puerarin (7.35 U/mg), as well as some activity with carminic acid and mangiferin, whereas its activity with the O-glycosides rutin and naringin was low. Remarkably, the specific activity of *Sc*POx measured for puerarin (7.35 U/mg) is much higher than for d-glucose (0.045 U/mg) or other monosaccharides, even though its concentration in the assay was significantly lower (0.2 mM as compared to 200 mM for the sugars).

Steady-state kinetic constants for different electron acceptor and donor substrates are summarized in Table 2. For determining apparent steady-state kinetic parameters of electron acceptors, we selected d-xylose as constant, saturating substrate, as it had the lowest Michaelis constant K_m_. Accordingly, we selected DCIP as saturating substrate when varying the concentrations of the electron donors for the determination of their kinetic constants. Since determined K_m_ values for all sugar substrates are far higher than expected, the resulting catalytic efficiencies are very low, ranging from 0.01 to 0.18 M^−1^·s^−1^, which is considerably lower than the catalytic efficiencies reported for *Ka*POx (10,000, 210 and 4400 M^−1^·s^−1^ for d-glucose, d-xylose and d-galactose, respectively) or fungal POx [6,12]. Substrate saturation could not be reached when determining the catalytic constants for d-glucose and d-ribose when using oxygen (air) as the electron acceptor. Hence, these data should be considered estimates. Steady-state kinetic parameters for C- and O-glycosides were not determined due to their poor solubility in water.

### 2.3. Stability Studies

**Figure 4:**
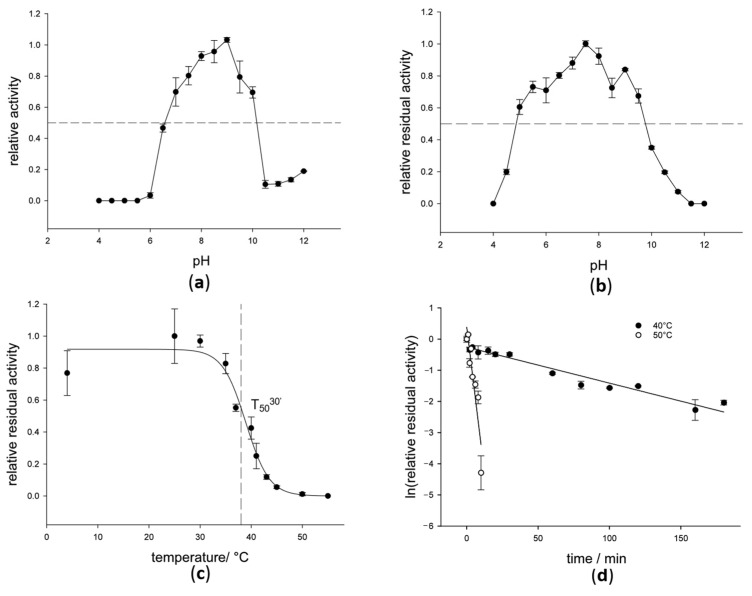
Enzyme stability explored with different approaches: (**a**) pH optimum profile for *Sc*POx at 30 °C. Areas in the plot that have more than 50% activity retained are indicated with a dashed horizontal line; (**b**) pH stability profile for *Sc*POx at 30 °C. Area in the plot that has more than 50% of activity retained is indicated with a dashed horizontal line; (**c**) T_50_^30’^ profile for *Sc*POx in 50 mM potassium phosphate buffer, pH = 6.5 and (**d**) half-life times at 40 and 50 °C in 50 mM potassium phosphate buffer, pH = 6.5. Measurements were done in triplicates and the mean value ± standard deviations are shown.

Analysis of the pH dependence of *Sc*POx activity revealed that the enzyme shows highest activity around pH 8–9 (Figure 4a). The enzyme retained >50% of its activity upon incubation in different buffers at 30 °C for 30 min within a pH range of 5–9.5 (Figure 4b). Thermostability of *Sc*POx was determined by measuring the T_50_^30’^ value: the temperature needed to inactivate 50% of the enzyme when incubated in 50 mM potassium phosphate buffer (KPP), pH = 6.5 for 30 min, which was found to be 38 °C (Figure 4c). Additionally, half-life times (t_1/2_) were measured at 40 and 50 °C (Figure 4d), resulting in 59 and 2 min, respectively. The thermodynamic stability of *Sc*POx was assessed by measuring its melting temperature (T_m_) using differential scanning calorimetry (DSC). Thermal unfolding displayed a single transition at 52 °C (50 mM KPP, pH = 6.5), corresponding to the temperature where 50% of the protein is in an unfolded state.

The effect of different ions and solvents on the activity was also investigated (Supplementary Materials Table S1). The measurements showed that *Sc*POx has a moderate tolerance to ions, as well as a high tolerance to denaturing agents and weak solvents. In summary, *Sc*POx has a moderate thermal stability and moderate tolerance to different environmental conditions (temperature, pH, composition of buffer).

### 2.4. Structural Model of ScPOx

The structure of *Sc*POx was modelled by using RoseTTaFold and evaluated by comparison with the crystal structures of fungal, tetrameric POx (PDB accession ID 1TT0) from *T. multicolor* (*Tm*POx), as well as monomeric FAD-dependent C-glycoside 3-oxidase (PDB 7DVE) from *M. trichothecenolyticum* (*Mt*CarA). The latter is phylogenetically more closely related to *Sc*POx but positioned in a separate clade of Actinobacteria, Micrococcales sequences (Figure 1). A sequence comparison of *Tm*POx, *Mt*CarA and *Sc*POx together with other previously characterized bacterial enzymes, *As*POx, *Ka*POx and pyranose oxidase from *Phanerochaete chrysosporium* (*Pc*POx) can be found in the Supplementary Materials Figure S3. Error estimates of the model are shown in the Supplementary Materials Figure S4.

**Figure 5:**
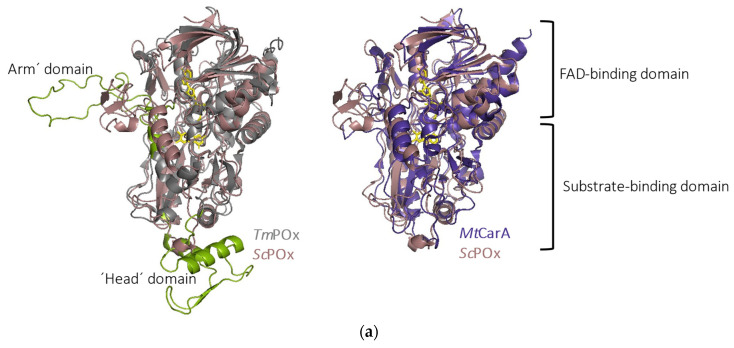

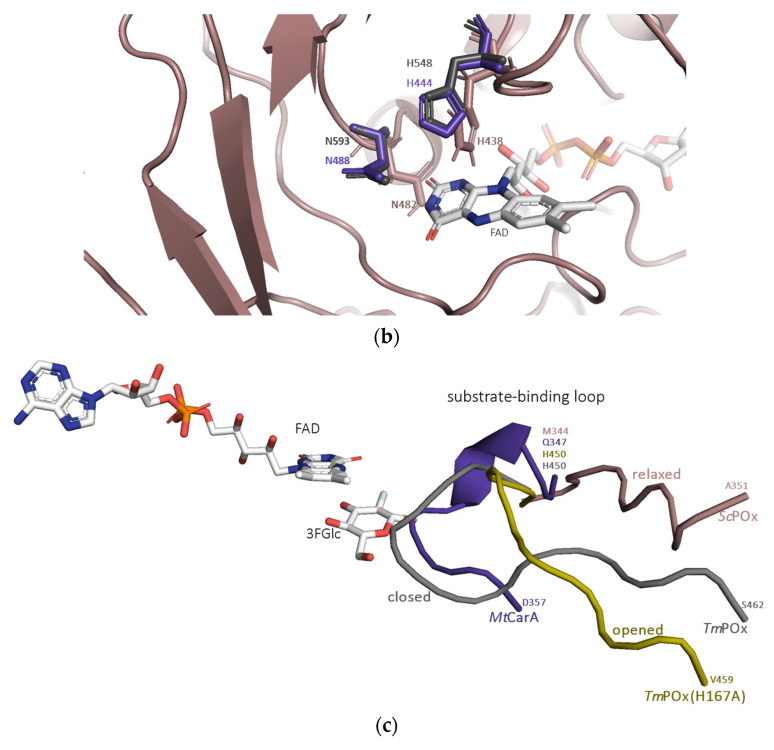
Structural model of pyranose oxidase from *Streptomyces canus* (*Sc*POx). (**a**) Superimposition of pyranose oxidase from *Trametes multicolor* (*Tm*POx) (grey, only one monomer shown) and *Mt*CarA from *Microbacterium trichothecenolyticum* (purple) with *Sc*POx (pink) in cartoon mode. The FAD molecule from the crystal structure of *Tm*POx or *Mt*CarA is depicted in yellow. Important oligomerization parts of the *Tm*POx structure are coloured green (“arm” residues 105–158 and “head” residues 368–430); (**b**) Comparison of the spatial positions of the catalytic His-Asp dyad of *Sc*POx (pink) with those of *Tm*POx (grey) as well as *Mt*CarA (purple). The FAD moiety is shown in light grey; (**c**) Comparison of different substrate-binding loop conformations of *Sc*POx (pink), *Mt*CarA (purple), *Tm*POx (grey) and *Tm*POx variant H167A (yellow). FAD and the 3-fluoroglucose molecule (from structure 2IGO) are shown in light grey.

Superimposition of the structures of *Tm*POx and *Mt*CarA with the model of *Sc*POx showed that *Sc*POx shares the overall tertiary fold of GMC enzymes with an FAD-binding domain and a substrate-binding domain (Figure 5a). Despite an amino acid identity of only 24.2% between *Sc*POx and *Tm*POx, and 34.3% between *Sc*POx and *Mt*CarA, the rmsd values for the Cα atoms were 1.48 Å for *Sc*POx and *Tm*POx (for 462 Cα atoms), and 1.19 Å for *Sc*POx and *Mt*CarA structures (for 468 Cα atoms). The main differences between *Tm*POx and *Sc*POx were found in the oligomerization loop that forms the “arm” of tetrameric *Tm*POx, as well as in the “head” domain of *Tm*POx, a domain of unknown function that is missing in bacterial POx sequences and interestingly also in *Pc*POx. Instead of a long “arm” loop, the *Sc*POx model shows a shorter loop structure that includes a small α-helix and two β-sheets. Highest error estimates were calculated for this loop in the *Sc*POx model (Supplementary Materials Figure S4), which indicates that it could be very flexible. All bacterial POx sequences lack the N-terminal region of *Tm*POx as well (Supplementary Materials Figure S3), which has been proposed to be involved in oligomerization as judged by the structure of this tetrameric enzyme [14].

The catalytic His-Asn pair is well preserved in all POx sequences (Supplementary Materials Figure S3). This pair is formed by H438 and N482 in the *Sc*POx active site. While the structural model shows a good agreement with the positioning of the His residue with the corresponding residues from *Tm*POx and *Mt*CarA, (H548 and H444, respectively), N482 shows a different conformation, namely a rotation by 95.7° compared to N593 and N488 from *Tm*POx and *Mt*CarA, respectively (Figure 5b). The active-site loop ^450^HRDAFSYGAVQQ^461^ of *Tm*POx was reported to form a gate to the active site and thus plays an important role in substrate binding [15]. This loop is highly dynamic in *Tm*POx and was found to be a switch between a closed (PDB 1TT0) and an open conformation (PDB 2IGO) upon interaction with a monosaccharide substrate [16]. A comparison of the conformation of the corresponding substrate-binding loop of the *Sc*POx model (M344-P355) with the open and closed conformation of the *Tm*POx loop shows that it is in a more relaxed conformation, restricting the access to the active site less than the loop in *Tm*POx (Figure 5c). This might also explain the differences in the reactivities and substrate range of *Sc*PO and *Tm*POx. Furthermore, the error estimates for the residues in the active-site loop (Supplementary Materials Figure S4) suggest a certain flexibility of the loop in ScPOx as well. The modelled position of this loop is also different from that of *Mt*CarA. Docking of carminic acid into the active site of *Mt*CarA showed that the aglycone of this substrate clashes with the active-site loop, hence a conformational rearrangement might be necessary for bacterial POxs as well to accommodate the bulky glycosides [13]. This will have to be answered by further structural studies, though.

## 3. Discussion

Pyranose oxidase was first described in 1968 in the basidiomycete *Spongipellis unicolor* (*Polyporus obtusus*) and was subsequently studied from several wood-decomposing fungi [3]. The first report on a bacterial POx was published in 2016, and only two bacterial enzymes have been characterized as pyranose oxidases so far—those from *K. aureofaciens* and *A. siccitolerans* [11,12]. Genome data provided a wealth of information on putative POx sequences of both bacterial and fungal origin, yet this wide sequence space is only poorly explored experimentally. A phylogenetic analysis of these putative sequences showed that bacterial pyranose oxidases form various distinct clades (Figure 1). One of these clades, containing sequences from Streptomycetales and Pseudonocardiales, clusters closely with the fungal POx sequences, and contains *Ka*POx as one of its members. *As*POx (as well as *Mt*CarA) belongs to a clade of Micrococcales sequences that is clearly separated from the above-mentioned clades. Our phylogenetic analysis revealed several additional clades of putative bacterial POx sequences that are completely unexplored to date. To gain a better understanding of biochemical properties of bacterial POxs, we selected the sequence of a member of a Streptomycetales clade, POx from *Streptomyces canus* (*Sc*POx), for a more detailed study to further explore the wide sequence space of bacterial POx.

*Sc*POx is phylogenetically closer to *As*POx and *Mt*CarA than to *Ka*POx and the fungal POxs, and this is also reflected in its properties. *Sc*POx is a monomeric enzyme with a molecular mass of 52 kDa (*As*POx, 54 kDa; *Mt*CarA, 55 kDa) whereas *Ka*POx is homodimeric (two subunits of 61 kDa each), and fungal POxs are typically homotetrameric (four subunits of ~65 kDa). The FAD is non-covalently attached in *Sc*POx as in *As*POx and *Mt*CarA, whereas it is tethered to a His in both *Ka*POx and fungal POxs. Interestingly, this FAD-binding His residue is well conserved even in bacterial POx sequences (Supplementary Materials Figure S3). It has been suggested that the STHW flavinylation motif of fungal POx sequences (GTHW in *Ka*POx) is needed for flavinylation and since it is not present in full in the bacterial sequences [11], the covalent attachment is not found. *Sc*POx was shown to oxidize monosaccharides that are typical POx substrates—d-glucose, d-galactose and d-xylose—albeit with catalytic efficiencies that are significantly lower, by up to four orders of magnitude, compared to values typically reported for *Ka*POx or fungal POxs (e.g., catalytic efficiencies for the substrate pair d-glucose/oxygen are ~0.2, 10,000 and 73,000 M^−1^ s^−1^ for *Sc*POx, *Ka*POx and *Tm*POx, respectively). This is mainly because these sugars show Michaelis constants in the molar range for *Sc*POx (Table 1), which is a strong indication that they are in fact not the natural substrates of *Sc*POx.

Recently, Kumano et al. [13] reported that bacterial enzymes closely related to POxs, termed FAD-dependent C-glycoside 3-oxidase (CarA), catabolize both various C-glycosides and O-glycosides, but not d-glucose [13]. They showed that these enzymes oxidize the glucose moiety of C-glycosides such as carminic acid, primarily at the C-3, but also at the C-2 hydroxyl group, which is in accordance with the regiospecificity of oxidation of fungal POxs, which preferentially oxidize at C-2, but can further oxidize C-2 oxidized d-glucose or d-galactose at C-3 as well [5,17]. In fact, the sequence of *Mt*CarA from *M. trichothecenolyticum* clusters with that of *As*POx (Figure 1) and shows sequence identity to this sequence and the sequence of S*cP*Ox (Supplementary Materials Figure S3), so “FAD-dependent C-glycoside 3-oxidase” and “pyranose oxidase” refer to enzymes of the same sequence space (homologs). Since we presumed that monosaccharides are not the natural substrates of *Sc*POx, we also tested various C- and O-glycosides as possible substrates. Based on the determined specific activities, some of these are in fact much better substrates than d-glucose for *Sc*POx, and we obtained the highest specific activity of 7.35 U/mg for the C-glycoside puerarin. Interestingly, *Mt*CarA from *M. trichothecenolyticum* and closely related enzymes from *A. globiformis* (*Ag*CarA) and *Microbacterium* sp. 5-2b (CarA) showed no activity with puerarin, the C8-glucoside of daidzein. Kumano et al. [13] stated that the position of the sugar moiety on the aglycon is important for activity, and that their CarA homologs strongly preferred C-6-glucosylated compounds, which we did not confirm for *Sc*POx. Hence, it seems that bacterial POxs can show activity on a range of different glycosides, and that the activity to oxidize glycosides is found in various bacterial POx clades. Adding to that the relatively low sequence identity shared between different POx clades, we can probably expect even more functional variation to occur in the hitherto unexplored clades of bacterial POxs.

C- and O-glycosides are naturally found in plants and are known to be metabolized by glycosyltransferases and glycoside hydrolases [18,19]. Intestinal microorganisms deglycosylate C-glycosides by a two-step reaction: an oxidation of the sugar moiety, albeit with NAD(H)-dependent oxidoreductases, and a subsequent enzyme-catalysed C-C bond-cleaving step [20]. It was recently suggested that CarA and its homologs—and hence probably also bacterial POxs—play a crucial role in the metabolism of C-glycosides in soil bacteria by catalysing the first step of an equivalent two-step reaction, however with oxygen-dependent enzymes [13]. An ancestor of pyranose oxidase could thus have mainly functioned for the metabolization of plant-derived, sugar-containing compounds such as the C- and O-glycosides. Certain bacterial pyranose oxidases, such as the one from *K. aureofaciens*, or fungal pyranose oxidases, which were suggested to have been acquired by horizontal gene transfer from bacteria, could then have evolved and specialized over time to oxidize lignocellulose-derived sugars such as d-glucose, d-xylose or d-galactose.

## 4. Materials and Methods

### 4.1. Chemicals, Solutions, Buffers

The *Escherichia coli* expression strain BL21(DE3) was ordered from New England Biolabs (Frankfurt, Germany). The media used for all bacterial cultures was Luria Bertani (LB) broth (10 g/L of peptone from casein, 5 g/L yeast extract, 5 g/L NaCl) with an addition of ampicillin (Roth; Karlsruhe, Germany) to its final concentration of 100 µg/mL. Lactose monohydrate for the induction of gene expression was from Roth. p-Benzoquinone (p-BQ) and ferrocenium hexafluorophosphate ([Fe(C_5_H_5_)_2_]PF_6_) were purchased from Sigma-Aldrich (St. Louis, MO, USA) and 2,6-dichlorophenolindophenol (DCIP) from Fluka (Buchs, Switzerland). Horseradish peroxidase and 10-acetyl-3,7-dihydroxyphenoxazine (Amplex Red) were produced by Sigma-Aldrich and Chemodex (St. Gallen, Switzerland), respectively. N- and O-glycosides were obtained from abcr (Karlsruhe, Germany), except for carminic acid, which was from Glentham Life Sciences (Corsham, UK). The following buffers were used for protein purification: “buffer A” for affinity chromatography (50 mM Tris-HCl, pH = 7.5, 150 mM NaCl, 5% glycerol, 30 mM imidazole), “buffer B” for affinity chromatography (50 mM Tris-HCl, pH = 7.5; 150 mM NaCl; 5% glycerol and 300 mM imidazole), “storage buffer” for storing purified proteins (50 mM Tris-HCl, pH = 7.5; 150 mM NaCl; 10% glycerol), and a size exclusion chromatography buffer (50 mM Tris-HCl, pH = 7.5; 150 mM NaCl). The “universal buffer” used for determining pH optima and pH profiles was Britton Robinson buffer (50 mM boric acid; 50 mM phosphoric acid and 50 mM acetic acid titrated to a desired pH value). All other solutions were of standard recipe unless otherwise stated.

### 4.2. Bacterial Pyranose Oxidase Genes and Expression

Ten different coding sequences from various phylogenetic clades of the bacterial pyranose oxidase phylogenetic tree were selected. These sequences, predicted to code for pyranose oxidases, originated from the following organisms: *Frankia alni* (Uniprot accession number Q0RGV3)*, Paenibacillus alvei* (S9TU08), *Domibacillus aminovorans* (*A0A177L2D9*), *Klebsiella pneumoniae* (A0A1S8Y799), *Rhizobium hainanense* (A0A1C3VGT0), *Deinococcus aerius* (A0A2I9D0D5), *Microbacterium testaceum* (A0A147F038), *Streptomyces canus* (A0A117Q443), *Geodermatophilus amargosae* (A0A1I6Z5L5) *and Pseudomonas frederiksbergensis* (A0A291AJ39). Coding sequences for all ten bacterial genes were ordered and cloned into the expression vector pET-21d+ between the restriction sites *Nco*I and *Hind*III by the company BioCat (Heidelberg, Germany). Gene codons were optimized for *E. coli.* All constructs carried a C-terminal His_6_ tag as well as ampicillin resistance. Calcium-competent *E. coli* strain BL21(DE3) was transformed with the constructs using the heat-shock transformation method. Cells were grown in 250 mL and 500 mL LB medium with ampicillin inoculated with overnight cultures diluted 1:90. For the expression of the recombinant genes, bacterial cultures were grown at 37 °C with agitation (130 rpm) until OD_600_ reached 0.6–1. After that, induction of gene expression was started by adding 10 mM lactose, and the temperature was decreased to 18 °C. Overexpression lasted for approximately 20 h, and cell pellets were collected by centrifugation (20 min, 5000 rpm, 8 °C, centrifuge Beckman Coulter Avanti J-26 XP, rotor JA-10 (Brea, CA, USA)). These cultivations yielded ~8.5 g of wet cell pellet per L of cell culture. Molecular properties of the recombinantly produced proteins were calculated by ProtParam (https://web.expasy.org/protparam/, accessed on 20 October 2019) [21].

### 4.3. Protein Purification

Cell pellets were resuspended in buffer A (1 mL of buffer per 1 g of wet cell pellet), and then disrupted by sonication with ultrasonic homogenizer Bandelin Sonoplus HD 60 (Berlin, Germany) at 120 V and 30% cycle for 5 min. Sonication was repeated 3 times with 5 min breaks on ice in between. The soluble cell extract was separated from cell debris by centrifugation (1 h, 25,000 rpm, 4 °C, centrifuge Beckman Coulter L1F 737, rotor 70Ti). Affinity chromatography was used to purify the His-tagged proteins from this crude cell extract, which was loaded onto a pre-equilibrated 5 mL HisTrap NP Ni Sepharose column (Cytiva; Marlborough, MA, USA) with flow rate of 2 mL/min. After washing the column with buffer A, elution was carried out with a linear increase to 100% buffer B during 15 min with the flow rate of 1 mL/min. When using methods such as SEC-SLS and DSC, size exclusion chromatography (SEC) was used as an additional purification step. Pooled fractions from affinity chromatography were loaded onto a 120-mL Superdex 75 (Cytiva) size exclusion column with the flow rate of 0.5 mL/min. The same flow rate was used to separate the proteins of interest from residual proteins. For both purification methods, fractions that were coloured bright yellow and showed absorption at 280 nm, as well as 389 and 450 nm, were pooled and concentrated/desalted using Amicon Ultra centrifugal filter units (MWCO 30 kDa). The purity of protein samples was checked by SDS-PAGE, and the presence of the His-tag was confirmed by Western blotting. Protein concentrations were determined with both the Bradford assay using commercially available bovine serum albumin (BSA; Thermo Scientific Pierce; Waltham, MA, USA) as standard and measuring absorbance at 280 nm (Agilent 8453 Diode Array spectrophotometer; Santa Clara, CA, USA). Both methods agreed very well and we used the mean values of these two independent measurements. Purified proteins were stored in “storage buffer” at −80 °C.

### 4.4. Analysis of Molecular Properties, Oligomeric State, and Protein Concentration

Routinely, the analysis of the approximate protein (subunit) mass was done by SDS-PAGE. The electrophoresis system used was from BioRad (Hercules, Clearwater, FL, USA), and appropriate precast gels were used (Mini-PROTEAN TGX Stain-Free Precast Gels, 4–16%). Preparation of protein samples as well as the electrophoresis procedure were done according to the manufacturer’s recommendations. Visualization was carried out in a GelDoc (BioRad). When analysing protein samples by Western blotting using penta-His-Tag monoclonal antibodies (Qiagen; Germantown, MD, USA), a published protocol was used [22]. To determine the oligomeric state of native proteins, analytical size exclusion chromatography coupled with right-angle light scattering (SEC-SLS) was carried out. SEC-SLS analysis was conducted on an OMNISEC multi-detector GPC/SEC instrument (Malvern Panalytical; Malvern, UK) equipped with refractive index, right-angle light scattering (RALS) and a UV/VIS diode array detector. Protein samples were applied to a Superdex S200 increase 10/300 GL column (Cytiva; Marlborough, MA, USA) maintained at 25 °C, using phosphate-buffered saline as an isocratic mobile phase at a flow rate of 0.5 mL/min. The injection volume varied between 20 and 100 μL. Protein concentrations were measured online by using the refractive index detector. The instrument was calibrated using commercially available BSA (2 mg/mL).

### 4.5. Spectroscopic Analysis and Identification of the Cofactor

The UV-Vis spectra of purified proteins (protein concentration of 20 mg/mL) were recorded between 250 and 500 nm with an Agilent 8453 Diode Array spectrophotometer at room temperature. Reduction of the protein upon incubation with 4 M d-xylose was performed in the anaerobic environment of a glove box equilibrated with N_2_ prior to measurements. The final concentration of O_2_ in the glovebox was <0.2%. Extraction of the cofactor was done as follows: 5% v/v trichloroacetic acid (TCA) was added to 20 mg/mL of protein sample. The solution was then incubated at 600 rpm and 25 °C for 1 h, and subsequently centrifuged at 13,000 rpm for 10 min. The supernatant was neutralized with solid Na_2_CO_3_ (Roth) to the final pH value of 7. The spectra of these supernatants were measured as described and additionally analysed by MALDI-TOF mass spectrometry. To this end, samples were spotted with the dihydroxybenzoic acid matrix on the MALDI target plate in various dilutions. MALDI-TOF mass spectrometry was performed with a Bruker Autoflex MALDI-TOF instrument in the positive ion reflection mode. Scans were recorded in the range of 500–2000 m/z. Manual data analysis was done by using the software flex-Analysis v 1.3 (Bruker; Billerica, MA, USA).

### 4.6. Kinetic Analysis

All activity assays were carried out with a PerkinElmer Lambda 35 spectrophotometer (Waltham, MA, USA). Apparent steady-state kinetic parameters for the electron acceptors p-BQ and DCIP were determined by varying the concentration of the electron acceptors while keeping the electron donor concentration constant (4 M d-xylose for *Sc*POx). d-Xylose was selected as the constant electron donor as it had the lowest Michaelis constant K_m_. Wavelengths and extinction coefficient values were used as previously described [22]. Steady-state kinetic constants for O_2_ were determined as reported [12].

Steady-state kinetic data for different electron donors were measured by varying the concentration of electron donors (50–2000 mM) while keeping the DCIP concentration constant (0.3 mM). When using oxygen (air) as the constant electron acceptor, the peroxidase-coupled assay with Amplex Red was used to measure hydrogen peroxide, with horseradish peroxidase and Amplex Red in concentration of 7.15 U/mL and 0.05 mM, respectively. Absorbance was measured at 560 nm with an extinction coefficient of 55.5 mM^−1^ cm^−1^ for Amplex Red. All assays were performed at 30 °C in triplicates using 50 mM potassium phosphate buffer, pH = 6.5. The concentration of *Sc*POx in the assays was 5.6 µg/mL. The amount of enzyme that oxidizes 1 μmol of substrate per minute was defined as one unit of enzymatic activity. The steady-state kinetic constants were calculated using the software SigmaPlot v 12 (Systat Software; San Jose, CA, USA). v_max_ and K_m_ values were determined using nonlinear least-square regression by fitting the observed data to the Michaelis-Menten equation:

(1) $$v\;=\frac{v_{\max}\left[S\right]}{K_{m}+\left[S\right]}$$

The turnover number k_cat_ was calculated using v_max_ values and the molecular mass of the *Sc*POx monomer.

### 4.7. Stability Measurements

All kinetic enzyme stability measurements were performed with an activity assay using 0.3 mM DCIP as electron acceptor as well as 600 mM d-glucose as an electron donor and were carried out as described in the Kinetic Analysis section. d-Glucose was preferred here, as d-xylose solutions of high concentrations (4 M) were very viscous and not well suitable for routine measurements, while 600 mM d-glucose was convenient for this purpose. pH optimum determinations (dependence of the activity on the pH value) were performed in 50 mM universal buffer from pH 4.5 to 12 with pH increments of 0.5. pH stability measurements were performed by incubating the enzyme at 30 °C for 30 min in universal buffer of a given pH value. After that, residual activity was measured using the standard setup for enzyme activity assays. The temperature of half inactivation, i.e., the temperature where activity drops to 50% of the initial value within a given time (T_50_^30’^), was measured by incubating the enzyme for 30 min at the certain temperature in a thermoblock, starting at 25 °C. After that, the residual activity was measured using standard assay conditions. The measurements were repeated in the range of 25 to 55 °C in incremental steps of 3 °C. Lastly, the half-life of activity (t_1/2_) was determined for 40 and 50 °C by incubating an enzyme sample at a set temperature in a thermoblock and varying the time of the incubation. Again, residual activity was measured using standard assay conditions. The data were fitted to the equation:

(2) $$\ln\left(\mathrm{relative}\;\mathrm{activity}\right)=\ln{\left(\mathrm{relative}\;\mathrm{activity}\right)}_{t=0}-k_{d}t\;$$

where k_d_ is a rate constant of deactivation. The half-life of activity was calculated with the following equation:

(3) $$t_{1/2}=\frac{\ln2}{k_{d}}$$

The effects of different ions and compounds were investigated by introducing the desired component into the solution and measuring the activity as described in the Section 4.6.

To determine the thermal transition temperature (melting temperature, T_m_), i.e., the temperature at which 50% of protein is in its denatured state, differential scanning calorimetry (DSC) was used (MicroCal PEAQ-DSC Automated, Malvern Panalytical). The enzyme sample (325 μL in 50 mM KPP, pH = 6.5) was heated from 20 to 85 °C, with increments of 1 °C/min. The raw data were fitted with the software MicroCal PEAQ-DSC v 2.2 (Malvern Panalytical; Malvern, UK), using the fitting algorithm “non-two state”.

### 4.8. Structural Modelling

RoseTTaFold (https://robetta.bakerlab.org/, accessed on 13 October 2021) [23] was used to determine a structural model of *Sc*POx. The best model, annotated as “Model 1”, was used for further structural analysis. The structural model was explored in the software PyMOL v 2.5.2, educational license (Schrödinger; New York, NY, USA).

## 5. Conclusions

Here, we report the biochemical characterization and structural analysis of a newly discovered pyranose oxidase (POx) from *Streptomyces canus* (*Sc*POx). Even though the phylogenetic position of its sequence shows its relation to the bacterial POx from *Arthrobacter siccitolerans* and FAD-dependent C-glycoside 3-oxidase from *Microbacterium trichothecenolyticum*, the kinetic characterization showed that *Sc*POx is not a typical POx, substrate specificity-wise, as it prefers C-glycosides over monosaccharides. We also confirmed that the structural model of *Sc*POx shares the typical architecture of GMC-oxidoreductases. Based on our findings, and the knowledge of numerous already-characterized fungal POxs and two bacterial POxs, we conclude that *Sc*POx represents yet another sub-type of POx, preferentially utilizing the C-glycoside puerarin as substrate, while potentially more sub-types can be found in the sequence space of bacterial POxs.

## Figures and Tables

**Table 1:** Activities of *Sc*POx for different monosaccharides at final concentrations of 200 mM and various C- and O-glycosides at final concentrations of 0.2 mM, measured with DCIP as electron acceptor at 30 °C in 50 mM potassium phosphate buffer, pH = 6.5 in triplicates.

Substrate		Specific Activity (U mg^−1^)
Monosaccharide	d-glucose	0.045 ± 0.001
	d-galactose	0.012 ± 0.000
	d-xylose	0.018 ± 0.000
	d-ribose	0.003 ± 0.000
	l-arabinose	0.006 ± 0.001
C-glycoside	Carminic acid	0.018 ± 0.000
	Mangiferin	0.126 ± 0.001
	Puerarin	7.35 ± 0.13
O-glycoside	Naringin	0.006 ± 0.001
	Rutin	0.001 ± 0.000

**Table 2:** Apparent steady-state kinetic constants for different electron acceptor and donor substrates of *Sc*POx. The parameters were determined at 30 °C in 50 mM potassium phosphate buffer, pH = 6.5 in triplicates.

Saturating Substrate	Varied Substrate	*v_max_* (U mg^−1^)	*k_cat_* (s^−1^)	*K_m_* (mM)	*k_cat_*/*K_m_* (M^−1^ s^−1^)
d-xylose (4 M) as e^−^ donor	p-BQ	0.63 ± 0.02	0.54	0.13 ± 0.02	41,540
	DCIP	0.11 ± 0.00	0.10	0.05 ± 0.01	2000
	O_2_	0.02 ± 0.00	0.02	0.06 ± 0.01	333
DCIP (0.3 mM) as e^−^ acceptor	d-glucose	0.42 ± 0.02	0.36	2060 ± 250	0.18
	d-galactose	0.08 ± 0.01	0.07	1007 ± 132	0.07
	d-xylose	0.10 ± 0.01	0.09	847 ± 107	0.10
	d-ribose	0.02 ± 0.00	0.02	1417 ± 175	0.01
	l-arabinose	0.05 ± 0.00	0.04	1507 ± 257	0.03
O_2_ (~0.25 mM) as e^−^ acceptor	d-glucose	>0.44 *	>0.38 *	>2000 *	>0.19 *
	d-galactose	0.17 ± 0.03	0.15	5230 ± 1050	0.03
	d-xylose	0.10 ± 0.00	0.09	1215 ± 184	0.07
	d-ribose	>0.01 *	>0.01 *	>2000 *	>0.005 *
	l-arabinose	0.26 ± 0.05	0.23	9500 ± 2100	0.02

* Substrate saturation could not be reached, thus, these parameters are estimates.

## Data Availability

The data presented in this study are available within the article and the Supplementary Materials.

## Supplementary Materials

The following supporting information can be downloaded at: https://www.mdpi.com/article/10.3390/ijms232113595/s1.
