# Supplementary material for: Biochemical Characterization of Pyranose Oxidase from Streptomyces canus—Towards a Better Understanding of Pyranose Oxidase Homologues in Bacteria
Source: Int J Mol Sci. 2022 Nov 6;23(21):13595. doi: 10.3390/ijms232113595 (PMC9659204; doi:10.3390/ijms232113595)
Supplement: Supplementary file 1 [file ijms-23-13595-s001.zip › ijms-1982448-supplementary.pdf]

Biochemical characterization of pyranose oxidase from *Streptomyces canus* – towards a better understanding of pyranose oxidase homologues in bacteria

Anja Kostelac [1, 2], Leander Sützl [1], Jolanta Puc [1], Valentina Furlanetto [3], Christina Divne [3], Dietmar Haltrich [1, *]

[1] Department of Food Science and Technology, University of Natural Resources and Life Sciences - BOKU, Vienna, Austria
[2] Doctoral Programme BioToP - Biomolecular Technology of Proteins, University of Natural Resources and Life Sciences - BOKU, Vienna, Austria
[3] School of Engineering Sciences in Chemistry, Biotechnology, and Health - CBH, KTH Royal Institute of Technology, Stockholm, Sweden
* Correspondence: dietmar.haltrich@boku.ac.at

Figure S1. SDS-PAGE analysis of *Sc*POx purified by two chromatographic steps – Ni-NTA affinity chromatography and size-exclusion chromatography. The protein bands are compared to the molecular mass standard Precision Plus Protein Standards (BioRad) (indicated with 'M') in order to assess the molecular mass. 1 – *Sc*POx sample after Ni-NTA affinity chromatography, 2 - *Sc*POx sample after size-exclusion chromatography.

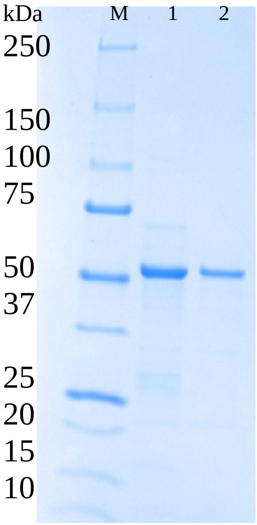

Figure S2. Determination of the monodispersity and molecular mass of purified *Sc*POx using analytical size exclusion chromatography coupled with right-angle light scattering, performed in 50 mM KPP buffer, pH=6.5. The profile shows the monomeric state with a precise molecular weight of 52.4 kDa.

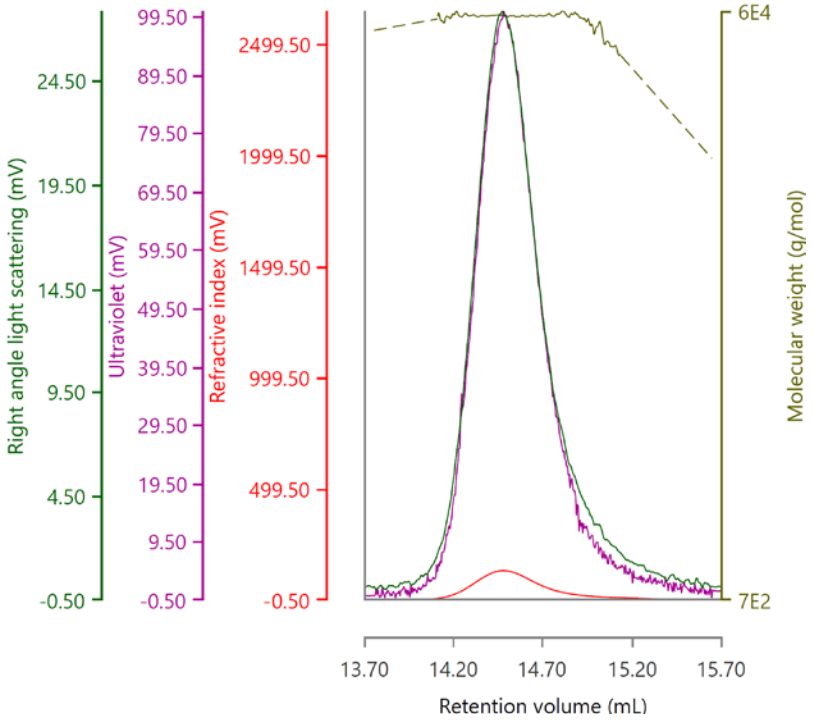

Figure S3. Multiple sequence alignment of fungal POxs from *Trametes multicolor* (*Tm*POx) and *Phanerochaete chrysosporium* (*Pc*POx), bacterial POxs from *Kitasatospora aureofaciens* (*Ka*POx), *Arthrobacter siccitolerans* (*As*POx) and *Streptomyces canus* (*Sc*POx) and bacterial FAD-dependent C-glycoside 3-oxidase from *Microbacterium trichothecenolyticum* (*Mt*CarA). The 'arm' and 'head' domain and N-terminal domain from *Tm*POx [8] are boxed as are the catalytic dyad (His and Asn) and the His residue involved in the covalent attachment of FAD in the two fungal representatives. The active-site loop is marked based on *Tm*POx structural studies [8]. All residues are coloured according to their physicochemical properties (Zappo colours).

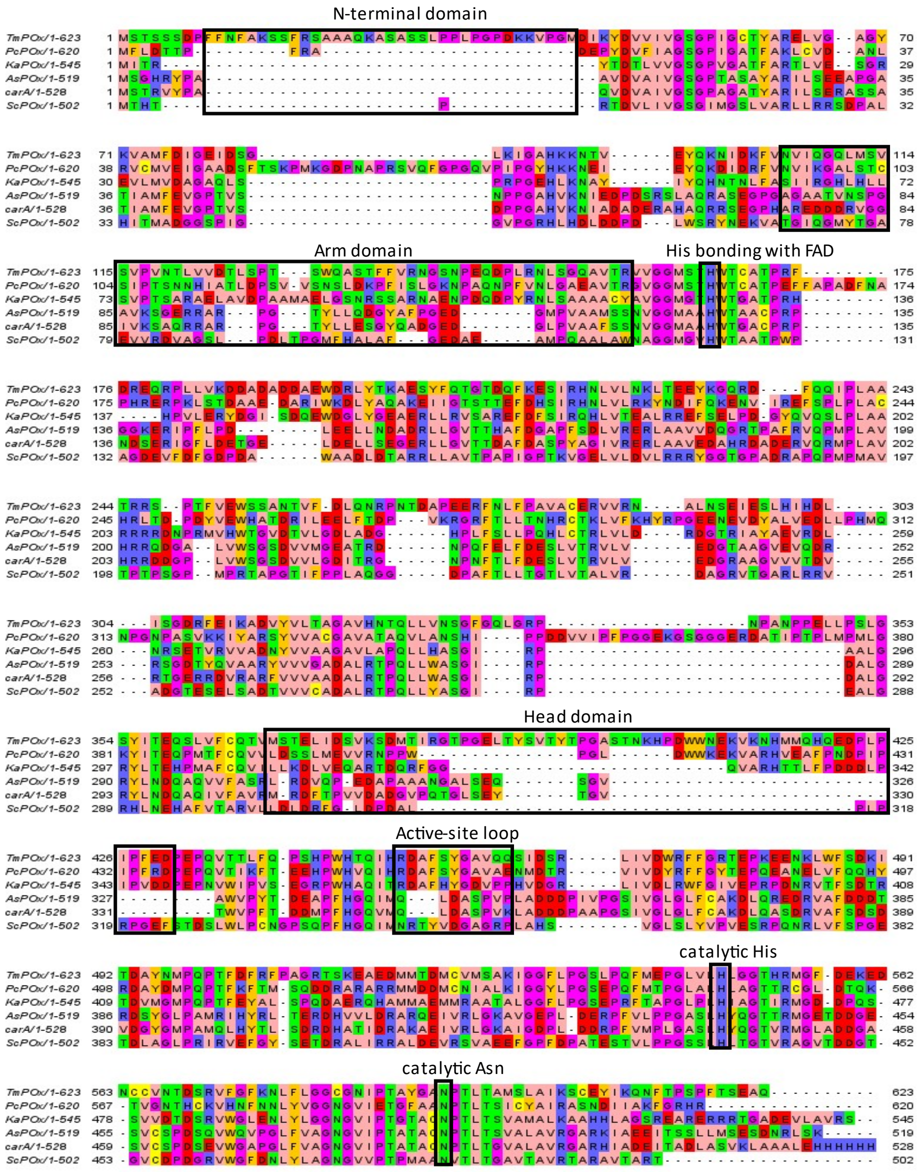

Figure S4. Error estimates per residue for the structural model of pyranose oxidase from Streptomyces canus. The model was calculated by using RoseTTaFold (https://robetta.bakerlab.org/) and the best model, annotated as ´Model 1´, was used for further structural analysis. These error estimates are based on structural variations within model clusters. The inset shows the detailed estimates for the active-site loop (M344-P355) discussed in the text.

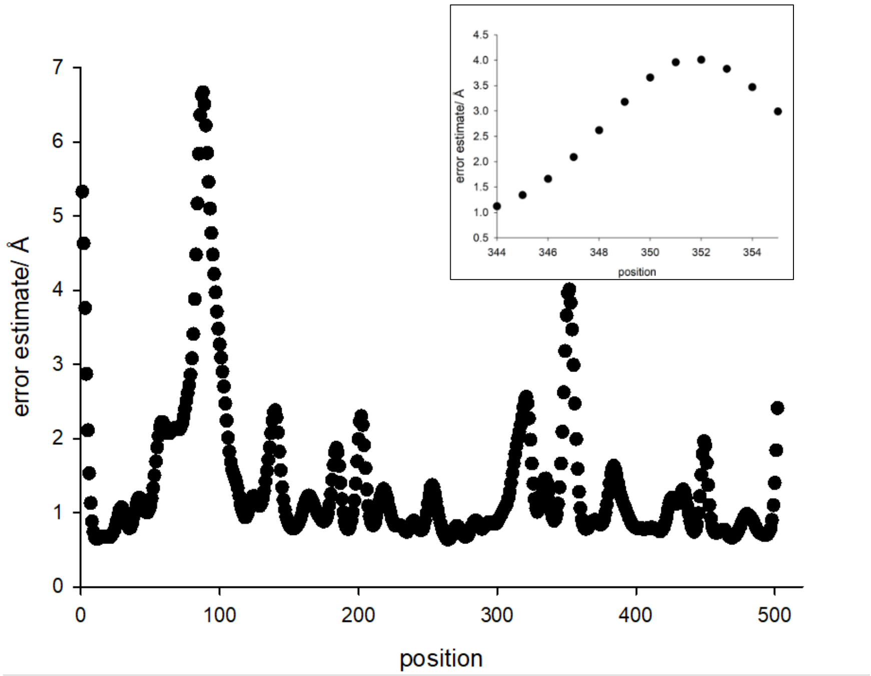

Table S1. Effect of different ions, denaturating agents and solvents on the activity of *Sc*POx. Values given are relative activities compared to standard assay conditions without any additive. All samples were measured at least in triplicates. n.d., not determined.

Ions	1 mM	5 mM	10 mM
NaCl	95.3 ± 2.7	91.7 ± 10.4	77.5 ± 9.8
KCl	92.1 ± 5.6	80.1 ± 3.4	88.2 ± 10.7
$MgCl_2$	100.9 ± 2.7	79.4 ± 5.9	64.4 ± 2.9
$CaCl_2$	85.8 ± 9.2	n.d.	n.d.
$ZnCl_2$	78.8 ± 2.5	n.d.	n.d.
$CoCl_2$	93.9 ± 4.3	n.d.	n.d.
$MnCl_2$	74 ± 5.0	n.d.	n.d.
$CuCl_2$	n.d.	n.d.	n.d.
$FeCl_3$	83.9 ± 8.9	n.d.	n.d.

Denaturating agents	1 mM	5 mM	10 mM
EDTA	97.0 ± 10.0	106.7 ± 5.9	131.5 ± 3.1
Urea	107.2 ± 4.9	100.3 ± 5.1	94.3 ± 4.2
Guanidine-Cl	95.0 ± 7.3	99.0 ± 2.2	100.9 ± 3.2
SDS	82.0 ± 5.8	n.d.	n.d.
DTT	0	0	0
β-mercaptoethanol	0	0	0

Solvents	1%	5%	10%
Glycerol	90.7 ± 3.9	104.1 ± 6.1	110.0 ± 6.1
Acetone	102.9 ± 9.8	100.2 ± 1.9	98.2 ± 3.4
Ethanol	92.9 ± 9.6	104.6 ± 6.1	103.9 ± 4.5
Tween 20	92.9 ± 4.6	54.8 ± 8.1	36.5 ± 4.3
